# Torque capacity of multidisc wet clutch with reference to friction occurrence on its spline connections

**DOI:** 10.1038/s41598-021-00786-6

**Published:** 2021-10-29

**Authors:** Marcin Bąk

**Affiliations:** Division of Hydraulics and Pneumatics, Faculty of Mechanical Engineering and Ship Technology, Gdansk University of Technology, ul. Gabriela Narutowicza 11/12, 80-233 Gdańsk, Poland

**Keywords:** Engineering, Materials science

## Abstract

In this article developed mathematical model that includes friction occurrence on spline connections is presented. The work also contains results of experimental research on torque capacity of multidisc wet clutch. These results are expressed as a function of contact pressure for different number of friction surfaces. Due to increased interest in research concerning multidisc wet clutches it is essential to determine impact of friction on fit connections on transmitted torque. Analytical calculations that include both known loss coefficient and assumed lack of friction on fit connections are compared to results of experiments. The paper contains detailed description of test stand and methodology of experiment. As a result of conducted tests it was found that correction coefficients known from literature are highly inaccurate. Measured values of torque indicate that transmitted torque reach significantly higher values. It was also revealed that after slippage appeared, the pressure plate usually moved in the direction of exerted clamping force, but movement in reversed direction also took place for some experiments. While movement corresponding to clamping force reached ca. 0.08 mm, in opposite direction amounted to 0.02 mm. Furthermore, studies presented that lapping of adjacent friction surfaces greatly affects differences between respective results obtained for a specific experiment.

## Introduction

Growth of application of automatic transmissions in agricultural and construction machinery drivetrains or car gearboxes that takes place lately, leads to increased interest in the assembly by both industrial companies and researchers. Although the gearbox contains many various components, one of its most important subassemblies is wet clutch. Its design and effects appearing in those clutches are common object of investigations, which are aimed to gain a knowledge about their influence on clutch performance, durability and overall efficiency of a gearbox.

Wet clutches are assemblies which elements are submersed in coolant fluid. They are responsible for transmitting drive, thus torque between two coaxially located shafts. In reference to multiple contact surfaces its torque capacity is greatly higher than typical dry clutch even if friction coefficient for wet contact faces is significantly smaller than for dry conditions^1^.

Both analytical and experimental research have been conducted in the past to investigate wet clutch behaviour in relation to thermal stresses, pressure and temperature distributions. Most of scientific paper about wet clutches corresponds to friction occurring between cooperative discs and influence of friction material properties on performance of the clutch and thermoelastic effects.

Zagrodzki formulated theoretical model that describes influence of stated above parameters and effects on a plate. Moreover, he found out that friction materials have a significant impact on clutch work and durability. Another part of his contribution to this scientific field refers to generation of hot spots in multidisc brakes or clutches. He highlighted importance of plate thickness, their elasticity modulus, and geometrical accuracy of friction surfaces on their creation^2–4^.

Holgerson investigated dynamic and thermal characteristics of clutch engagement in presence of drive torque. He found out that transmitted torque during engagement process is highly affected by drive torque presence. As a result of his research it was shown that drive torque has influence on many parameters, for example on temperature of fluid and discs^5,6^. Fatima examined relation of shaft inertia and its stiffness on friction characteristics and durability of a clutch. Experimental results revealed that possibility of shudder occurrence grows with duty cycles. Another important conclusion is that growth of assembly mass moment of inertia might have positive impact on potential torque oscillation, thus more comfortable performance from the driver’s point of view^7^.

Yang et al. investigated behaviour of multidisc lubricated clutch during short-term engagement both numerically and experimentally. Their research indicates that pressure and temperature distributions on friction surfaces are varied. Moreover, contact pressure on both friction surfaces of the disc are different^8^. Abdullah et al. studied influence of friction plate thickness on contact pressure on friction surfaces of multi-disc dry clutch. They discovered that disc thickness greatly affects dry clutch thermoelastic characteristic and that contact pressure magnitude grows significantly for thinner plates^9^.

Osinski showed estimation of torque capacity in reference to number of friction faces, its dimensions etc. His model implies that the engaged clutch transmits less torque compared to results obtained by simple analytical equation like friction area multiplied by mean values of pressure and radius^10^. Firstly, it is because of friction appearing on spline joints between a shaft and friction discs and separator discs and a hub. Secondly, friction plates sustain considerable deformations due to insufficient stiffness of a blocking disc as well as thermal deformations caused by heat generated during engagement and disengagement of the clutch.

In mathematical formula, an impact of mentioned above factors on transmitted torque is expressed as a coefficient, which value decreases for higher number of discs that forms the clutch. Though variety of studies were already conducted, there is still a lack of information about influence of connection type, dimensions, number of discs etc. on such coefficient. Therefore, it is reasonable to seek for new information referring to aforementioned effects.

The article aims to explore the influence of number of friction surfaces on torque capacity of a wet clutch. Also, the objective of the study is to analytically investigate importance of friction forces appearing on splines on torque transmitted by a clutch. The paper also intends to compare experimentally evaluated values of maximum transmitted torque to values calculated analytically with known formula. With reference to aims of the paper, the article consists of a developed mathematical model, as well as results of series of performed experiments. The model takes into consideration friction forces appearing on spline connections. Conducted experiments were performed for various number of friction surfaces. Additionally, obtained results of loss coefficient are compared to values of losses proposed by Osinski, highlighting error between two of them.

## Mathematical model of multidisc clutch

### Known mathematical model

**Figure 1:**
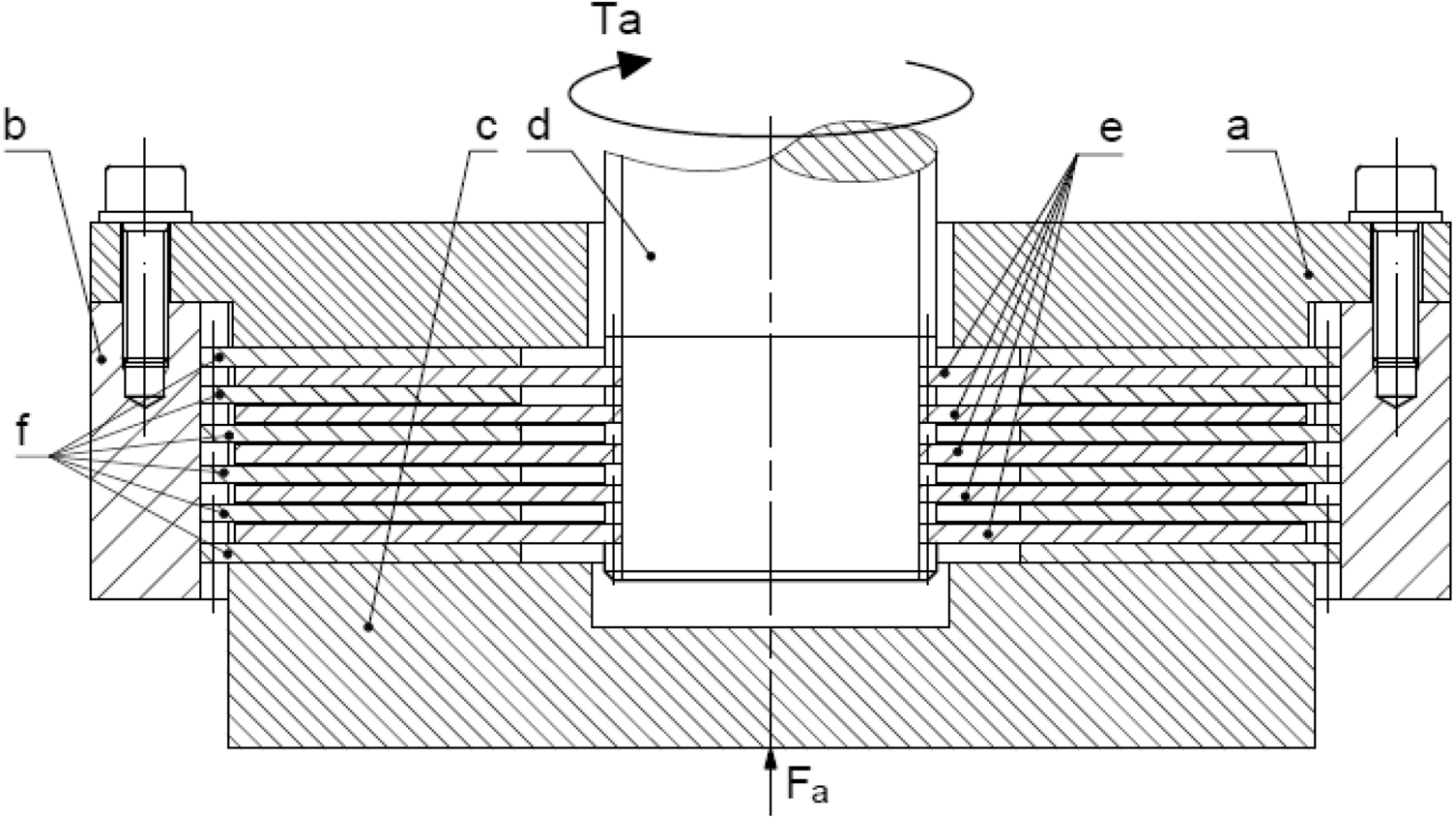
Simplified sectional view of clutch assembly: a—blocking plate, b—hub, c—pressure plate, d—shaft, e—friction discs, f—separator discs, F_a_—axial force, Ta—torque transmitted by a clutch.

One of currently known mathematical model of torque transmitted by multidisc wet clutch is based on friction transmitted by single, annular area multiplied by number of friction interfaces n^11,12^. In the Fig. 1 sectional view of a typical multidisc clutch is shown, which was used to illustrate the subject.

**Figure 2:**
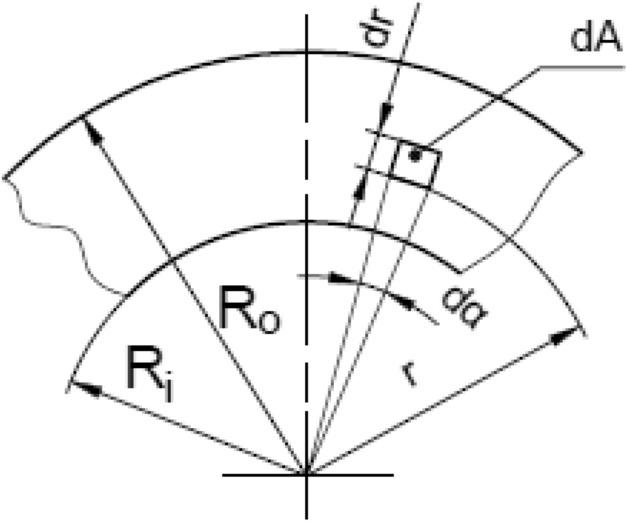
Presentation of annular contact area^13^.

Analytical torque Ta that can be transmitted by a fully engaged wet clutch, which contains n friction surfaces, can be calculated with expression:

1 $${\text{Ta = r}}_{{\text{m}}} \cdot {\text{n}} \cdot {\upmu } \cdot {\text{F}}_{{\text{a}}}$$

where ${\upmu }$ defines friction coefficient for a pair formed by friction and separator discs [-], ${\text{r}}_{{\text{m}}}$ is defined as mean radius of the annular area:

2 $${\text{r}}_{{\text{m}}} { = }\frac{2}{{3}}\frac{{{\text{R}}_{{\text{o}}}^{{3}} {\text{ - R}}_{{\text{i}}}^{{3}} }}{{{\text{R}}_{{\text{o}}}^{{2}} {\text{ - R}}_{{\text{i}}}^{{2}} }}$$

where R_o_ and R_i_ refers to outer and internal radius of annular, friction area, as shown in Fig. 2.

The friction area can be calculated as:

3 $${\text{A = }}\uppi \cdot {\text{(R}}_{{\text{o}}}^{{2}} {\text{ - R}}_{{\text{i}}}^{{2}} {)}$$

### Mathematical model proposed by Osinski

Due to inevitable friction occurrence in contact pairs between hub and separator discs and a shaft and friction discs Osinski implemented correction coefficient k_i_ into Eq. (1). This led to following equation:

4 $${\text{Ta}}_{{2}} {\text{ = k}}_{{\text{i}}} \cdot {\text{r}}_{{\text{m}}} \cdot {\text{n}} \cdot {\upmu } \cdot {\text{F}}_{{\text{a}}}$$

The values of the k_i_ coefficient proposed by Osinski are presented in the Table 1.

**Table 1:** Values of coefficient k_i_^10^.

Number of friction surfaces n	3	4	5	6	7	8	9	10	11
k_i_	1	0.97	0.94	0.91	0.88	0.85	0.82	0.79	0.76

Although specified values of k_i_ coefficient provide some information on losses regarding to friction, it is not known how they relate to for example different spline types, number of splines types etc. Therefore, application of the coefficient in design calculation might lead to substantial errors.

### Proposed mathematical model

As noted in previous chapter currently known mathematical models often refer to thermoelastic effects occurring on friction surfaces and to friction materials characteristics. For studies of number of friction on torque transmitted by a wet or dry clutch or brake an analytical model was developed. The model contains references to known expressions describing for example torque transmitted by one contact surface of a clutch or force distribution in spur gears, while the main purpose is to investigate impact of the friction forces on clutch performance.

**Figure 3:**
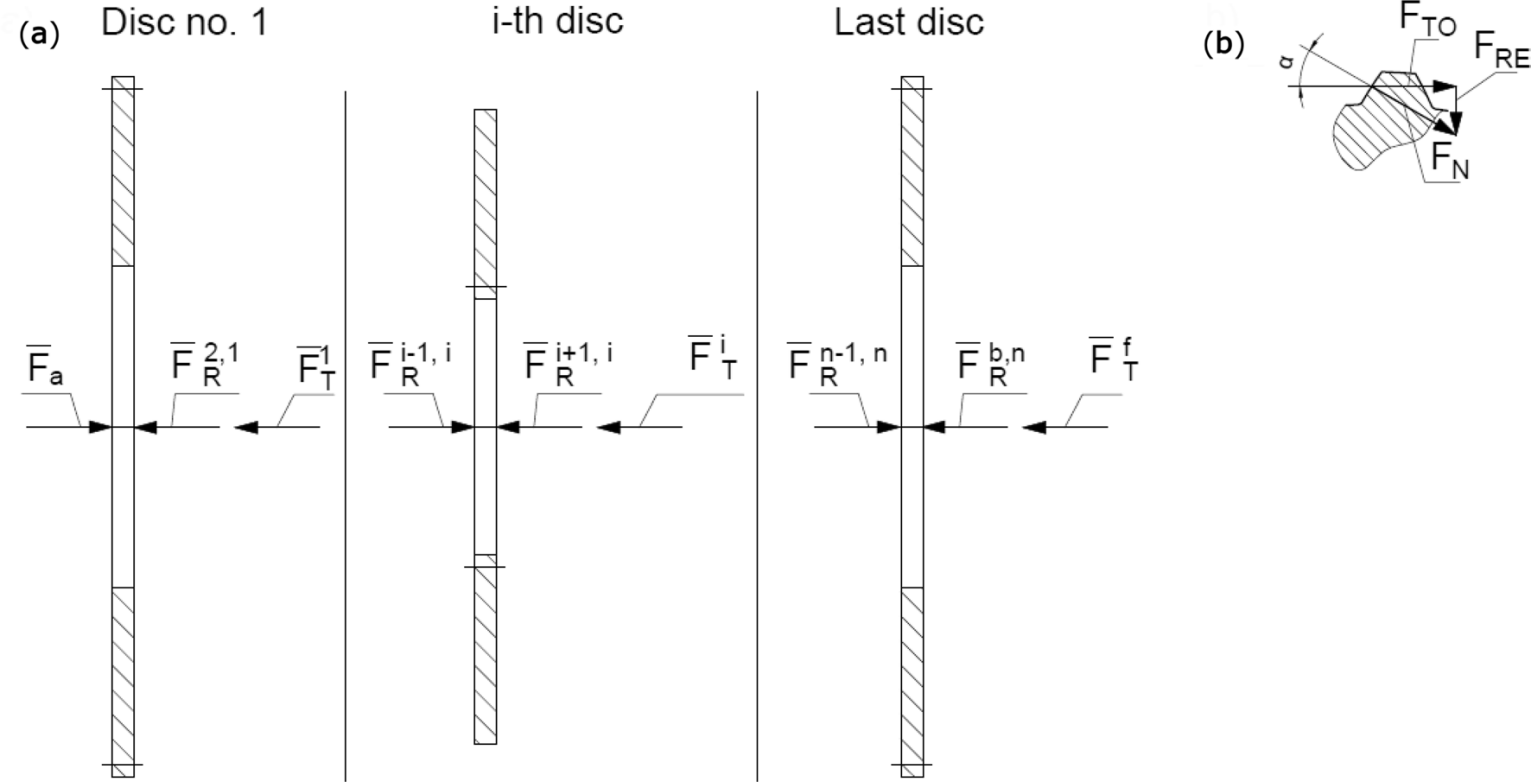
Physical model: (**a**) for three specified plates, (**b**) forces distribution in spline’s teeth: ${\text{F}}_{{\text{a}}}$—applied axial force to clutch package, ${\text{F}}_{{\text{R}}}^{{\text{i + 1,i}}}$—reaction force of disc i + 1 (or blocking disc) acting on disc i, ${\text{F}}_{{\text{T}}}^{{\text{i}}}$—friction force acting on disc a, ${\text{F}}_{{\text{N}}}$—normal force acting on a single tooth, ${\text{F}}_{{{\text{RE}}}}$—radial component of force ${\text{F}}_{{\text{N}}}$, ${\text{F}}_{{{\text{TO}}}}$—tangential component of force ${\text{F}}_{{\text{N}}}$.

Three chosen discs from the assembly shown in Fig. 1 with every force that acts on these respective discs are presented in the Fig. 3. The selected discs include:

- First disc with external spline, placed next to pressure plate,
- i-th disc, placed in the middle part of the clutch assembly,
- Last disc that have a common surface with the blocking plate when the clutch is engaged.

**Figure 4:**
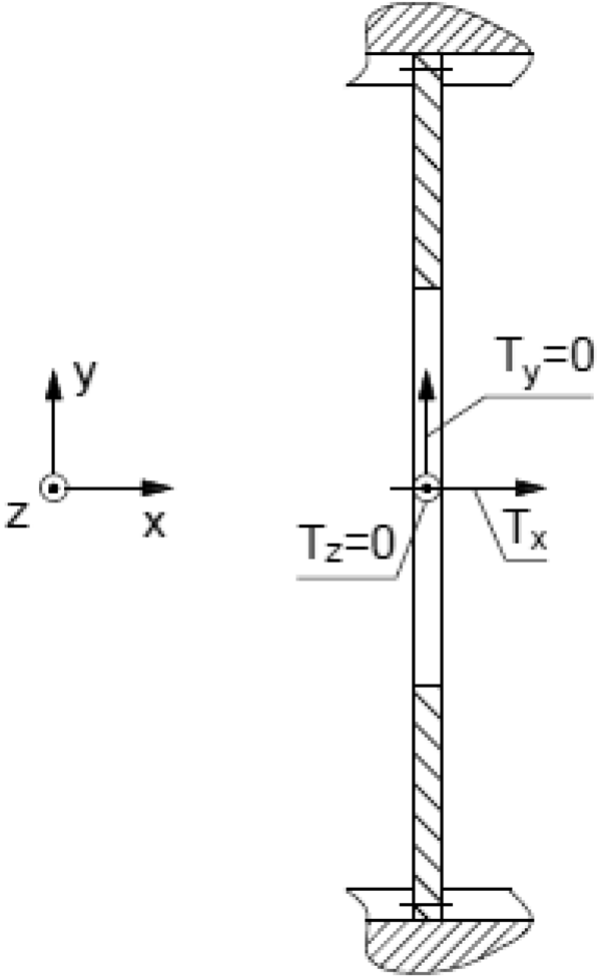
Distribution of moments acting on a single disc with external spline curvature.

The presented model in Fig. 3a consists simplifications that refer to omitted effects of thermal deformations and their influence on pressure distribution on friction surfaces, as well as friction on spline connections^14^. Due to those assumptions moments in axes perpendicular to axis of the clutch are equal to zero, as shown in the Fig. 4, which presents mentioned moments as T_z_ and T_y_. Consequently, it results in omission of possible nonparallel orientation of friction surfaces of a certain disc.

Expression (5) describes equilibrium of forces acting on disc no. 1 and holds references to both information mentioned above and Fig. 2, while formula (6) corresponds to i-th disc.

5 $${\text{F}}_{{\text{a}}} {\text{ = F}}_{{\text{R}}}^{{{\text{j1}}}} {\text{ + F}}_{{\text{T}}}^{{1}}$$

6 $${\text{F}}_{{\text{R}}}^{{{\text{ki}}}} {\text{ = F}}_{{\text{R}}}^{{{\text{l1}}}} {\text{ + F}}_{{\text{T}}}^{{\text{i}}}$$

Action and reaction forces ${\text{F}}_{{\text{R}}}^{{\text{i + 1,i}}}$ known also as Newton’s third law of motion between two adjacent elements are the sum:

7 $${\text{F}}_{{\text{R}}}^{{\text{i + 1,i}}} {\text{ = F}}_{{{\text{hp}}}} {\text{ + F}}_{{{\text{mc}}}}$$

where force ${\text{F}}_{{{\text{hp}}}}$ generated by pressure of the fluid that fills closed gaps between asperities^15^, force ${\text{F}}_{{{\text{mc}}}}$ exerted on discs as a result of mechanical contact between asperities of adjacent discs.

If relative rotational speed between discs that form closed gaps is equal to 0, then the viscous friction also equal to 0, which means that the clutch or brake is full engagement^16,17^. Having in mind that friction force is proportional to normal force acting on a surface, it is essential to reduce the hydrostatic force. Therefore, application of permeable friction materials is reasonable, due to absorption capacity and relatively high values of friction coefficient for a contact pair formed by for example paper based material and steel^10,18^.

Every friction surface has annular area A, created between two circles (Fig. 2). Torque transmitted by multidisc wet clutch could be calculated according to following expression:

8 $${\text{Te = }}\mathop \sum \limits_{{\text{k = 1}}}^{{\text{n}}} \mathop \int \limits_{{0}}^{{{2}\uppi }} \mathop \int \limits_{{{\text{R}}_{{\text{i}}} }}^{{{\text{R}}_{{\text{o}}} }} \frac{{{\text{F}}_{{\text{R}}}^{{\text{k, k + 1}}} }}{{\text{A}}}\upmu \cdot {\text{r}}^{2} \cdot {\text{dr}} \cdot {\text{d}}\upalpha$$

where k—amount of friction surfaces (minimum one and maximum n), r—distance between axis of a plate and elementary area dA (Fig. 2),

Substituting area A with formula (3) and completing mathematical operations in Eq. (8) leads to expression:

9 $${\text{Te = }}\frac{{2}}{{3}}{\uppi } \cdot {\upmu } \cdot \left( {{\text{R}}_{{\text{o}}}^{{3}} {\text{ - R}}_{{\text{i}}}^{{3}} } \right) \cdot \frac{{1}}{{{\uppi } \cdot \left( {{\text{R}}_{{\text{o}}}^{{2}} {\text{ - R}}_{{\text{i}}}^{{2}} } \right)}}\left( {{\text{n}} \cdot {\text{F}}_{{\text{a}}} { - }\mathop \sum \limits_{{\text{k = 1}}}^{{\text{n}}} {\text{(n - k + 1)}} \cdot {\text{F}}_{{\text{T}}}^{{\text{k}}} } \right)$$

It is assumed that friction surfaces are without grooves. If the grooves appeared, effective area on which axial force exerts would be noticeably smaller^19^.

The general relations between friction forces acting on selected tooth of spline (Fig. 3) are following:

10 $${\text{F}}_{{\text{N}}} { = }\frac{{{\text{F}}_{{{\text{TO}}}} }}{{{\text{cos}}\upalpha }}$$

11 $${\text{F}}_{{{\text{RE}}}} { = }\frac{{{\text{F}}_{{{\text{TO}}}} }}{{{\text{cot}}\upalpha }}$$

Tangential force ${\text{F}}_{{{\text{TO}}}}$ that acts on a certain tooth depends on number of teeth *z* that transmits summary torque T_*s*_ and pitch diameter ${\text{d}}_{{\text{p}}}$ of the manufactured spline and is calculated from formula:

12 $${\text{F}}_{{{\text{TO}}}} { = }\frac{{{\text{T}}_{{\text{s}}} }}{{\frac{{{\text{d}}_{{\text{p}}} }}{{2}} \cdot {\text{z}}}}$$

Torque T_*s*_ represents sum of friction torque transferred by both friction surfaces of the respective disc.

Previously mentioned formula that describes dependency between friction and normal force is given below:

13 $${\text{F}}_{{\text{T}}}^{{\text{k}}} { = }\upmu _{{{\text{sp}}}} \cdot {\text{F}}_{{\text{N}}}$$

where ${\upmu }_{{{\text{sp}}}}$ is a friction coefficient appearing on spline connection.

Combining formulas (10, 13) with expression (9) leads to following equation:

14 $${\text{Te = }}\frac{{2}}{{3}} \cdot {\uppi } \cdot {\upmu } \cdot {\text{p}}_{{{\text{av}}}} \cdot {\text{n}} \cdot \left( {{\text{R}}_{{\text{o}}}^{{3}} {\text{ - R}}_{{\text{i}}}^{{3}} } \right) \cdot {\text{(1 - B)}}$$

where B is loss coefficient corresponding to friction occurrence on spline connections and is defined by:

15 $${\text{B = }}\frac{{1}}{{{\text{n}} \cdot {\text{F}}_{{\text{a}}} }} \cdot \mathop \sum \limits_{{\text{k = 1}}}^{{\text{n}}} {\text{(n - k + 1)}} \cdot {\text{F}}_{{\text{T}}}^{{\text{k}}} {)}$$

and ${\text{p}}_{{{\text{av}}}}$ is a mean pressure appearing on friction surface when friction effects on spline connections are neglected:

16 $${\text{p}}_{{{\text{av}}}} { = }\frac{{{\text{F}}_{{\text{a}}} }}{{{\uppi } \cdot \left( {{\text{R}}_{{\text{o}}}^{{2}} {\text{ - R}}_{{\text{i}}}^{{2}} } \right)}}$$

Although above presented expression (15) is dependent on friction forces, assess of particular components would require iterative calculations for real assembly. Therefore, more convenient way to calculate torque transmitted by clutch is determine the coefficient of losses B from experimental data.

In order to visualize the differences between experimental torque Te and torque that could be transmitted if there would not be any loss on connection between friction discs and the shaft, as well as separator discs and the hub calculated with expression (1), the coefficient t was introduced and defined as:

17 $${\text{t = }}\frac{{{\text{Te}}}}{{\frac{{2}}{{3}}{\uppi p}_{{{\text{av}}}} {\upmu }_{{{\text{max}}}} {\text{n(R}}_{{\text{o}}}^{{3}} {\text{ - R}}_{{\text{i}}}^{{3}} {)}}}$$

where ${\upmu }_{{{\text{max}}}}$ is maximum static friction coefficient. Values of friction coefficients ${\upmu }_{{{\text{max}}}}$, ${\upmu }_{{{\text{sp}}}}$ would be different for both wet and dry clutch.

In order to compare dimensions of the contact surfaces between various designs of clutches the ratio i is defined as:

18 $${\text{i = }}\frac{{{\text{R}}_{{\text{i}}} }}{{{\text{R}}_{{\text{o}}} }}$$

In cars and heavy machinery the value of i is usually between 0.6 and 0.8^1,10^.

## Object of research

**Figure 5:**
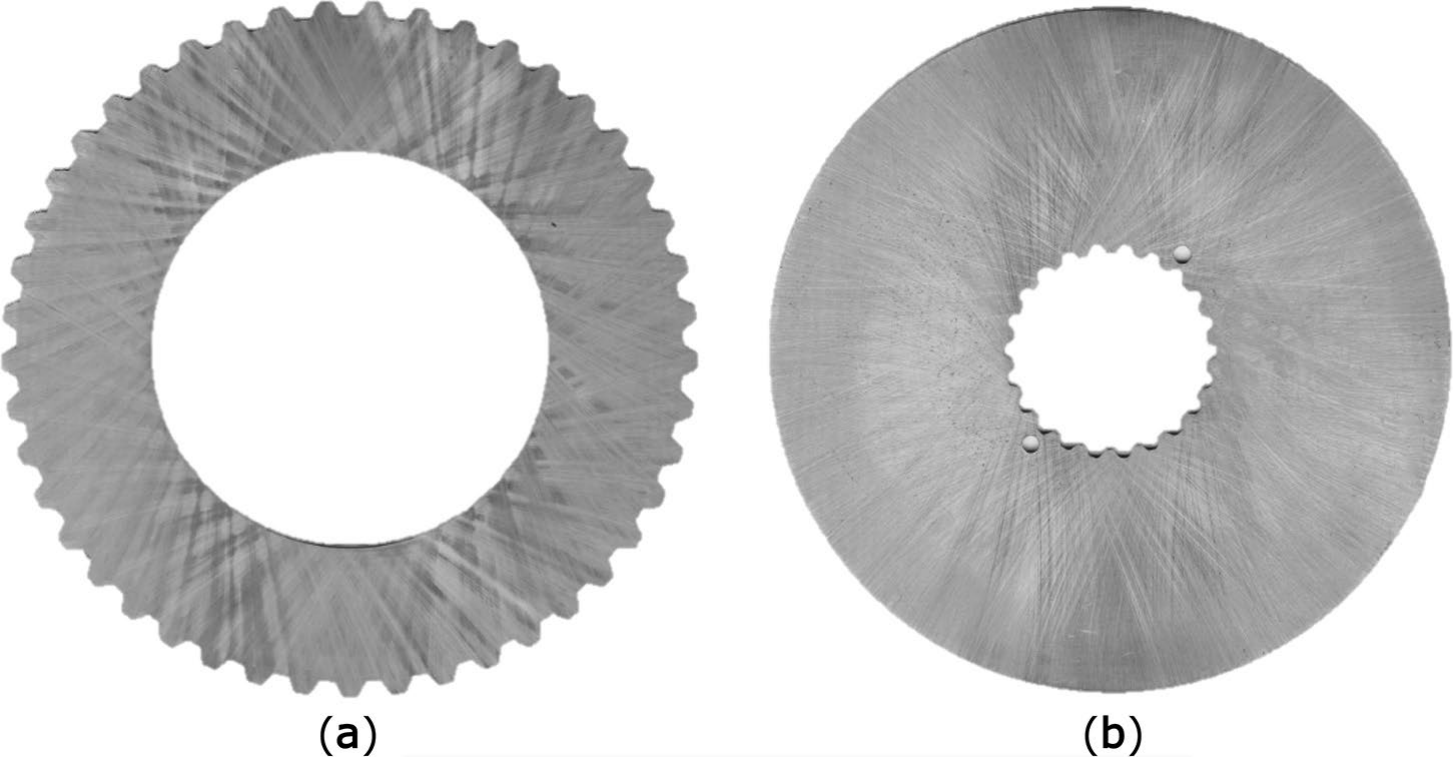
Clutch discs: (**a**) separator disc, (**b**) friction disc.

Object of research was composed of one variation of friction and separator discs. The chosen discs contain separator discs with internal friction radius R_i_ equal to 80 mm and friction discs which outer radius is equal to 115 mm. Both types of discs had thickness of 1.5 mm at the beginning of experiments and were made from steel S355J2. In the Fig. 5 unused discs are shown. Discs hardness were below 180 HB, thus their deformations of friction surfaces and potential deformations of splines ought to be more intensive. The contact surfaces of every disc were grinded and their initial roughness were below Ra = 1.25 μm. Designed flatness of both friction faces was limited to 0.02 mm. The same value was allowed as parallelism of those surfaces to each other. Designed discs were manufactured with involute splines according to standard DIN 5480 with a pressure angle equal to 30°. Friction discs with internal spline have 22 teeth and module and pitch diameter equal to respectively 1.5 and 33 mm, while the separator discs have 45 teeth with module equal to 3 mm and pitch diameter calculated to 135 mm. Dimensions of discs were chosen arbitrarily, based on manufacturers’ catalogues. As a result, teeth numbers were chosen arbitrarily. Both friction and separator discs were made without any grooves on their contact faces.

## Test stand

**Figure 6:**
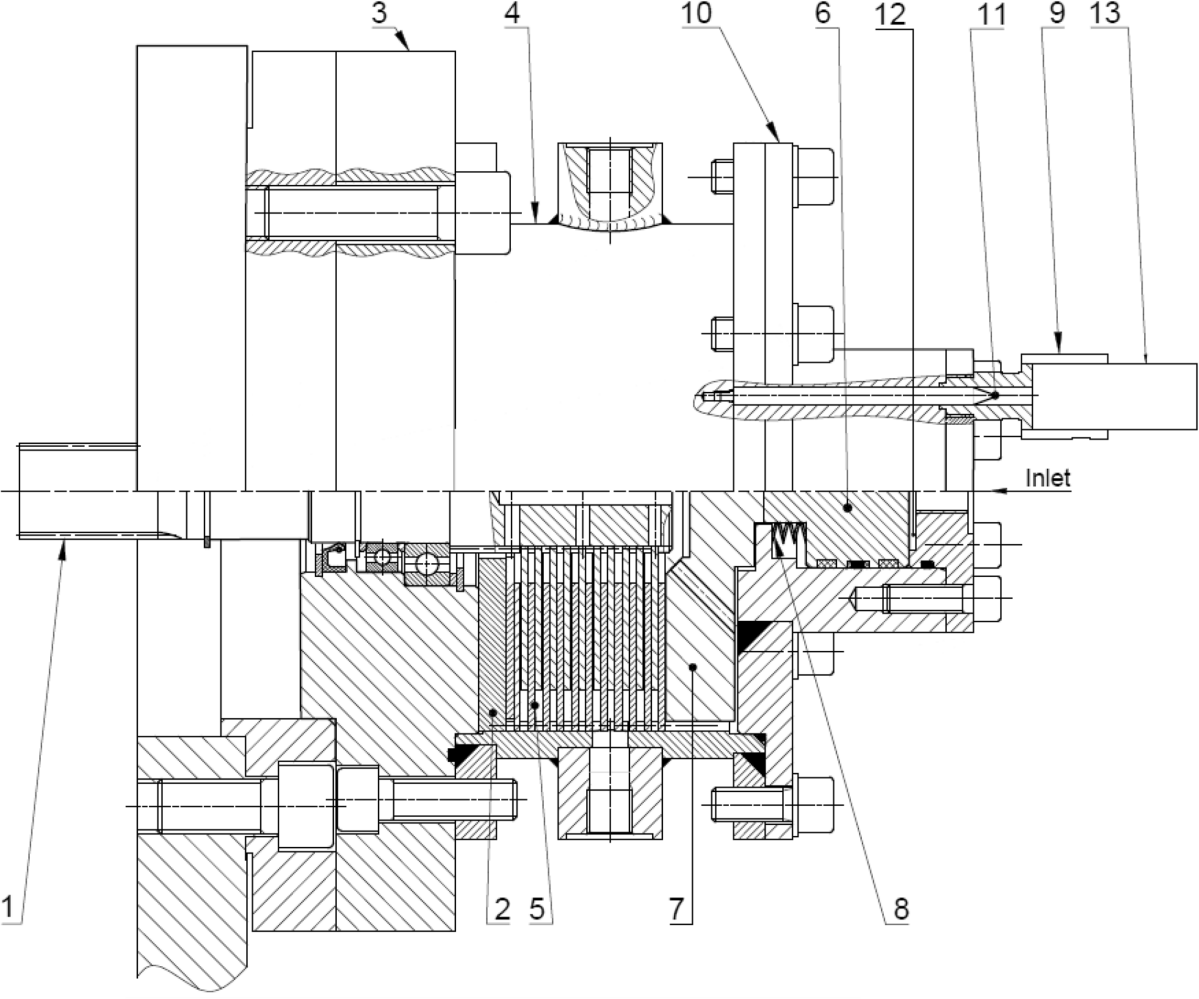
Half section view of multidisc wet clutch testing device^13,20^: 1—shaft, 2—blocking plate, 3—cover, 4—hub (housing), 5—friction and separator discs, 6—piston, 7—pressure plate, 8—package of disc springs, 9—position sensor holder, 10—cover, 11—position sensor tappet, 12—hydraulic actuator chamber, 13—displacement sensor.

A test stand was designed and built to provide the possibility of conducting several various types of experiments. A half section view of a multidisc wet clutch testing device is shown in Fig. 6. It could be used to study torque capacity, drag torque, durability of discs package, as well as engagement and disengagement time for various number of discs and their dimensions. Therefore, versatility of the test stand is its most significant asset.

The multidisc wet clutch testing device works as a multi-disc brake, where the hub 4 is stationary, connected to foundation, while the shaft 1 rotates with friction discs. Separator discs are mounted in the hub, alternately with friction discs. The torque transmitted by the clutch depends on axial force generated by the hydraulic actuator, thus pressure in chamber 12, number of discs 5 and their dimensions. Axial force is applied by the piston 6 to the pressure plate 7. If the axial force occurs, it clamps friction and separator discs 5, hence the clutch is engaged.

**Figure 7:**
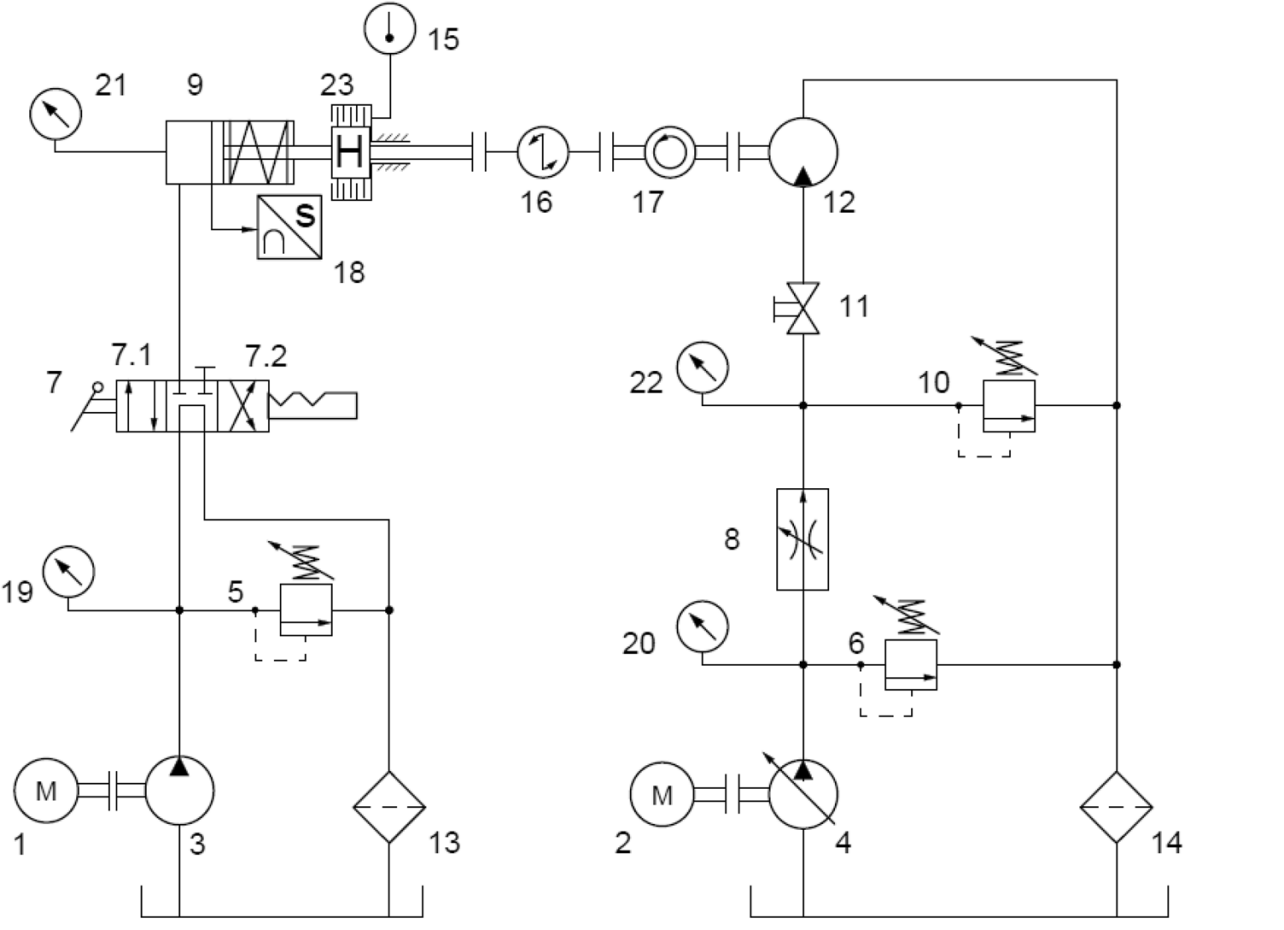
Hydraulic circuit^13,20^: 1, 2—electric motor, 3, 4—pumps, 5, 10—relief valves, 6—pressure valves, 7—4/3 directional valve, 8—flow control valve, 9—hydraulic actuator, 11—ball valve (shut-off valve), 12—hydraulic motor, 13, 14—filters, 15—temperature transducer, 16—torque transducer, 17—angular velocity sensor, 18—displacement sensor, 19–22—pressure transducers, 23—clutch.

The multidisc wet clutch testing device is driven by hydraulic motor, while hydraulic actuator is supplied by hydraulic circuit presented in Fig. 7. The hydraulic system contains two separate subsystems, one for supplying hydraulic actuator 9 and the second one for hydraulic motor 12^21^. The subsystems could work independently to each other due to application of two pumps 3, 4 and separate electric motors 1, 2 that drive the pumps.

Axial force exerted to the tested discs depends on pressure (measured with pressure transducer 21, Fig. 7) in hydraulic actuator 9 (Fig. 7) chamber 12 (Fig. 6), deflection of springs package 8 (Fig. 6) and friction force between piston sealing and guiding rings and cylinder of the actuator. It is widely known that pressure in an actuator chamber 12 (Fig. 6) have a noticeable impact on friction force, hence adjustment of pressure relief valve 5 (Fig. 7) affects effective force generated by the actuator 9 (Fig. 7)^22^.

To control rotational speed of hydraulic motor 12 (Fig. 7) during disengagement period adjustable two port flow control valve 8 (Fig. 7) was used. The valve was connected in high-pressure line of hydraulic motor, thus the subsystem worked as meter-in system. The value of torque transmitted from the motor to the brake depends on pressure measured by transducer 22 (Fig. 7) at the inlet port of the motor and is adjustable by relief valve 10 (Fig. 7).

**Figure 8:**
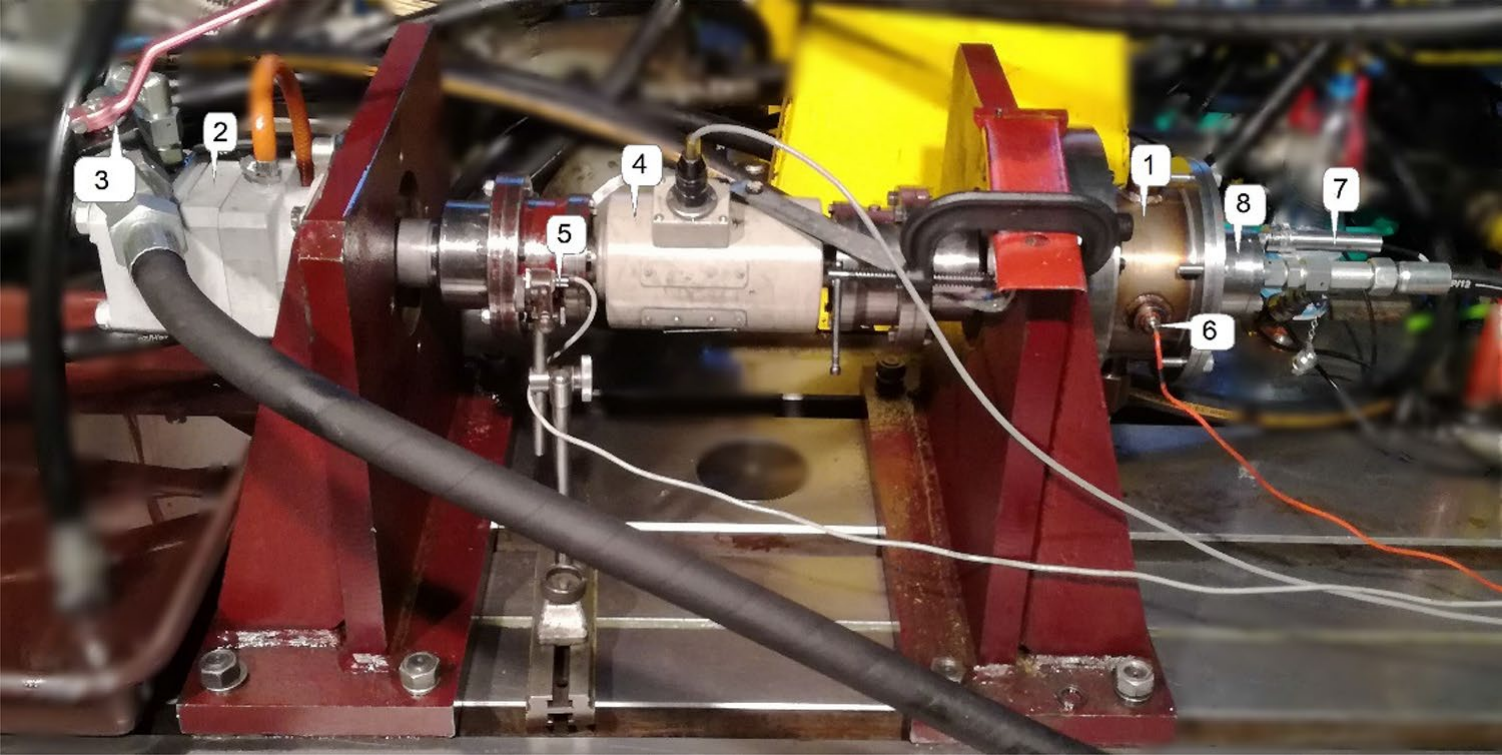
Test stand: 1—multidisc wet clutch testing device, 2—hydraulic motor, 3—shut-off valve, 4—torque transducer, 5—inductive sensor, 6—temperature sensor PT100, 7—displacement sensor, 8—hydraulic actuator.

Figure 8 presents view of complete, assembled test stand, which is driven by hydraulic motor. In the centre of the figure there is a torque transducer that couples shaft of the motor with the shaft of the pump.

The designed test stand enables experiments with range of parameters shown in the Table 2. It is possible to extend the scope of the experimental ability if another hydraulic motor would be used^20^. For example usage of high speed hydraulic motor could result in increased maximum rotational speed possible to achieve. Therefore, it could be possible to study drag torque with higher relative rotational speed between friction and separator discs.

**Table 2:** Parameters of test stand^13^.

Physical quantity	Range	Unit
Drive shaft angular velocity	0–500	rpm
Torque	0–400	Nm
Normal force	0–31,000	N
Maximum outer diameter D_o_	134	mm
Minimum internal diameter D_i_	36	mm

It is noticeable that the test stand allows to conduct experiments with ratio i even less than 0.3. Also, test stand enables to examine various friction lining materials.

Several different transducer were used during test to acquire all the necessary data. The measuring devices are shown in the Table 3. To record every analog signal generated by transducer measuring device Hydac HMG 4000 was used. Frequency of recording data was set at 1 kHz. To obtain rotational speed impulses generated by inductive sensor were counted. Then, knowing time step between subsequent impulses it was possible to calculate current value of angular velocity, thus rotational speed by dividing the angle between two detection objects by time step.

**Table 3:** Transducer used during experiments.

No.	Physical quantity	Device	Class	Range	Maximum error
1	Pressure	Pressure transducer	0.5	0–40 MPa	0.2 MPa
		Pressure transducer	0.6	0–40 MPa	0.24 MPa
		Pressure gauge	0.2	0–40 MPa	0.08 MPa
2	Torque	Torque transducer HBM	0.2	500 Nm	1.01 Nm
3	Rotational speed	Inductive sensor- Sick IME08	–	667 rpm	–
4	Temperature of oil	PT100	–	From − 50 to 400 °C	0.5 °C at 40 °C
5	Displacement of pressure disc	PSz	–	± 2.5 mm	–

In order to estimate axial force as accurately as possible it is obligatory to take into consideration all the forces mentioned in section “Mathematical model of multidisc clutch”.

The effective axial force F_a_ applied to the pressure plate is calculated as:

19 $${\text{F}}_{{\text{a}}} {\text{(p,s) = F}}_{{\text{p}}} \left( {\text{p}} \right){\text{ - F}}_{{\text{s}}} {\text{(s)}}$$

where F_s_(s) is force generated by package of springs as a function of their deflection s [N], F_p_(p) is normal force exerted by pressure measured with transducer 21 (Fig. 7) that acts on piston of the actuator reduced by friction force [N].

**Figure 9:**
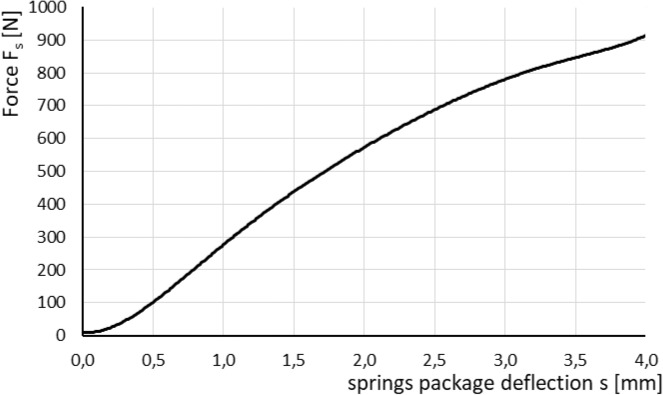
Characteristic of package of disc springs^13^.

Figure 9 shows relation between force F_s_ and total deflection s of all springs. The characteristic is a result of an approximation of experimental data gained by three repeated series of springs assembly characteristic determination.

The experimental characteristic of force F_s_ exerted by the deflected springs can be described by following formula:

20 $${\text{F}}_{{\text{s}}} \left( {\text{s}} \right){ = - 2}{\text{.9}} \cdot {\text{s}}^{{6}} { - 38}{\text{.063}} \cdot {\text{s}}^{{5}} { + 197}{\text{.57}} \cdot {\text{s}}^{{4}} { - 515.09} \cdot {\text{s}}^{{3}} { + 658}{\text{.13}} \cdot {\text{s}}^{{2}} { - 38}{\text{.596}} \cdot {\text{s + 9}}{.847}$$

**Figure 10:**
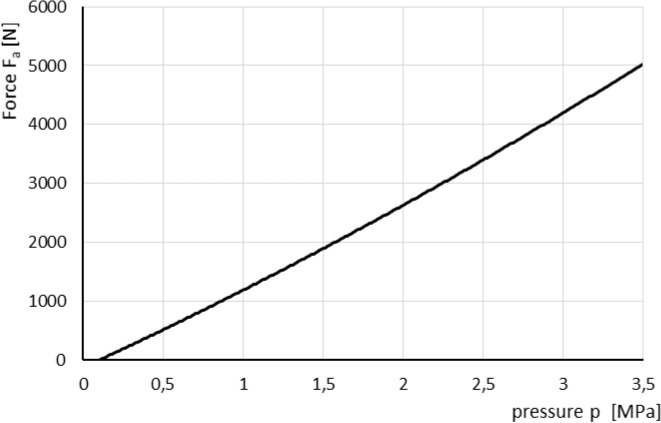
Characteristic of axial force applied to the piston^13^.

The same approach compared to described above method was used to determine relation between pressure in piston chamber and generated force F_a_. Results are presented in Fig. 10.

The experimental characteristic of force F_p_ can be described by following formula:

21 $${\text{F}}_{{\text{p}}} \left( {\text{p}} \right) = 0.6306p^{{2}} + 125.17p - 123.7$$

## Methodology of experiments

**Figure 11:**
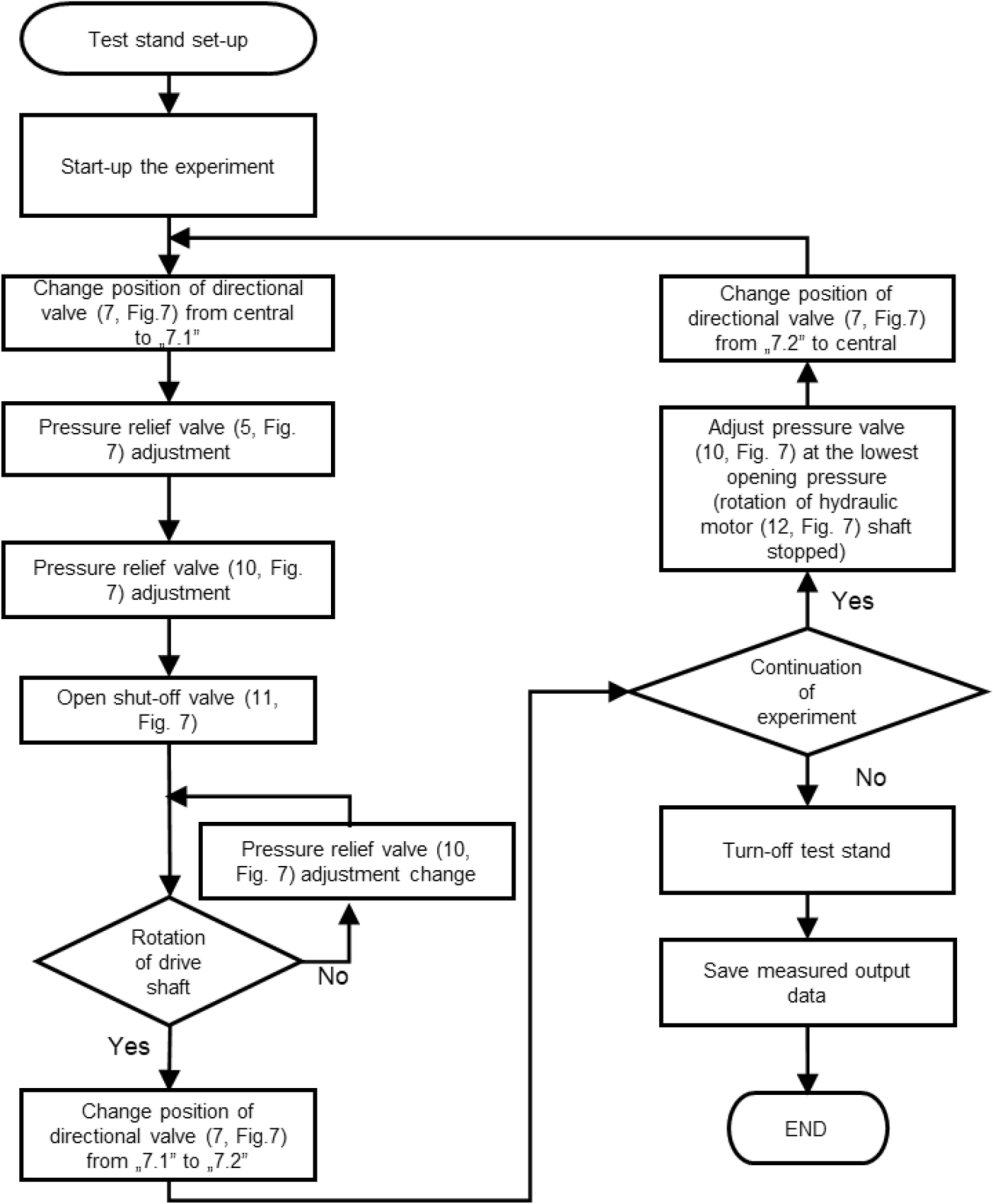
Block diagram of experiment methodology.

Methodology of performing tests is shown as a block diagram in Fig. 11. The block diagram contains references to hydraulic circuit shown in the Fig. 7 and illustrates process of maximum torque capacity determination. While first experiments were carried out for 18 friction surfaces, subsequent tests were performed for reduced number of friction surfaces i.e. 16, 14, 12, 10. Multidisc wet clutch device (Fig. 6) was disassembled between experiments conducted for varied number of friction surfaces to allow change of number of friction and separator discs. Removed friction and separator discs were substituted by distance discs. Their thicknesses were determined to maintain virtually the same thickness as the removed discs. They were mounted between cover (3, Fig. 5) and blocking plate (2, Fig. 5). Set-up process involved adjustment of relief valve (10, Fig. 7), controlling pressure at inlet of hydraulic motor, at its lowest opening pressure, while the shut-off valve was closed (11, Fig. 7). After start-up stage axial force was applied to friction and separator discs, therefore clutch was engaged. The axial force was dependent on pressure (measured with pressure transducer 21, Fig. 7) in actuator chamber. Afterward shut-off was open and pressure relief valve (10, Fig. 7) adjustment was increasing until the slippage occurred. Increasing pressure corresponds to higher torque transmitted by the clutch. Following tests for the same number of friction and separator discs were performed for the same or changed axial force. Great importance was pointed out to period of time occurring after slippage when the drive shaft rotated. First, short time between slippage and retraction of pressure plate is essential, due to large amount of heat generated when the relative motion occurs with presence of clamping force. This effect, known as two-body abrasive wear could result in critical damages on friction surfaces^23–25^. Thus, immediately after spotted rotation of motor shaft position of directional valve was changed. Secondly, to adequately represent conditions in real wet clutches or brakes, drive shaft rotated at relatively low rotational speed for dozens of seconds. It had been done to ensure that whole contact surfaces were submersed in lubrication fluid. Therefore, on every friction surface friction can be considered as equal to friction at wet environment.

## Results of experiments

### Transmitted torque by various number of discs

**Figure 12:**
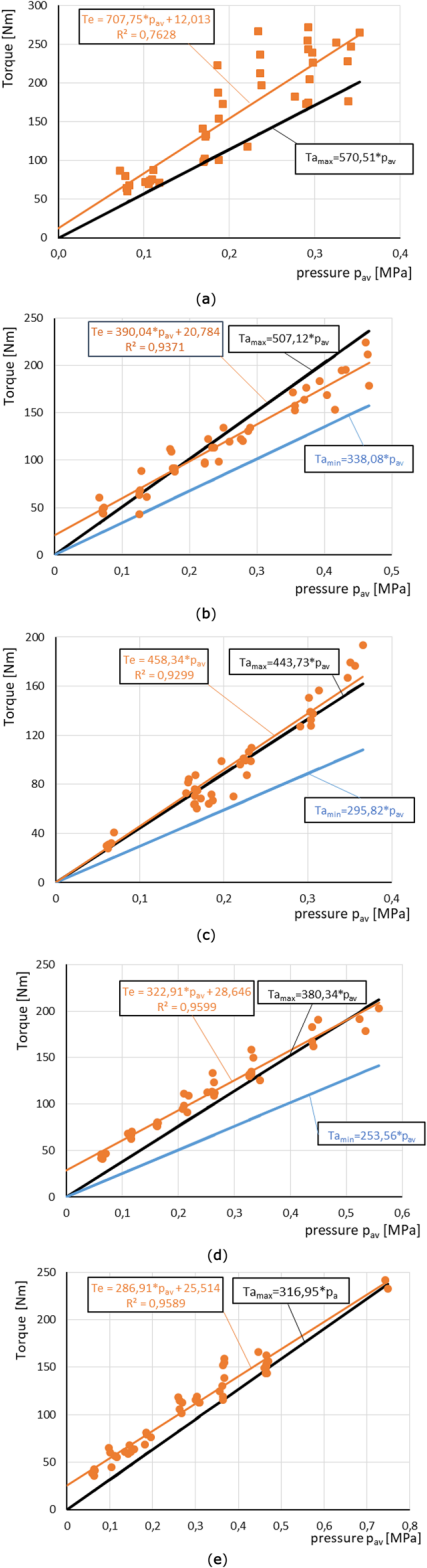
Comparison of torque transmitted by a clutch package formed by discs with different thicknesses as a function of mean value of contact pressure: D_i_ = 80 mm, D_o_ = 115 mm. Number of friction surfaces: (**a**) 18; (**b**) 16; (**c**) 14; (**d**) 12; (**e**) 10.

Plots showed in Fig. 12 depict obtained characteristics Te of transmitted torque in relation to number of friction surfaces. In contrast to experimental results and trend lines, noted as Te, maximum Ta_max_ and minimum Ta_min_ torque characteristics calculated with expression (1) are also presented on respective graphs. Te characteristics were created based on the acquired data. Ta_max_ curves relates to analytically obtained value of transmitted torque for maximum value of friction coefficient μ = 0.12. On the contrary, Ta_min_ refers to torque transmitted by the clutch when assumed static friction coefficient is equal to μ = 0.05^1^. Both values of friction coefficient refer to wet environment. If the best-fit line reached higher values than Ta_max_, then curve Ta_min_ was omitted on the graphs. Mentioned above functions are linear interpretations of torque transmitted by respective number for the highest and lowest magnitudes of static friction coefficient with omission of lost coefficient^1,10^. Interpretation of values of y-intercept for functions Te based on experimental data is given in the next chapter.

**Figure 13:**
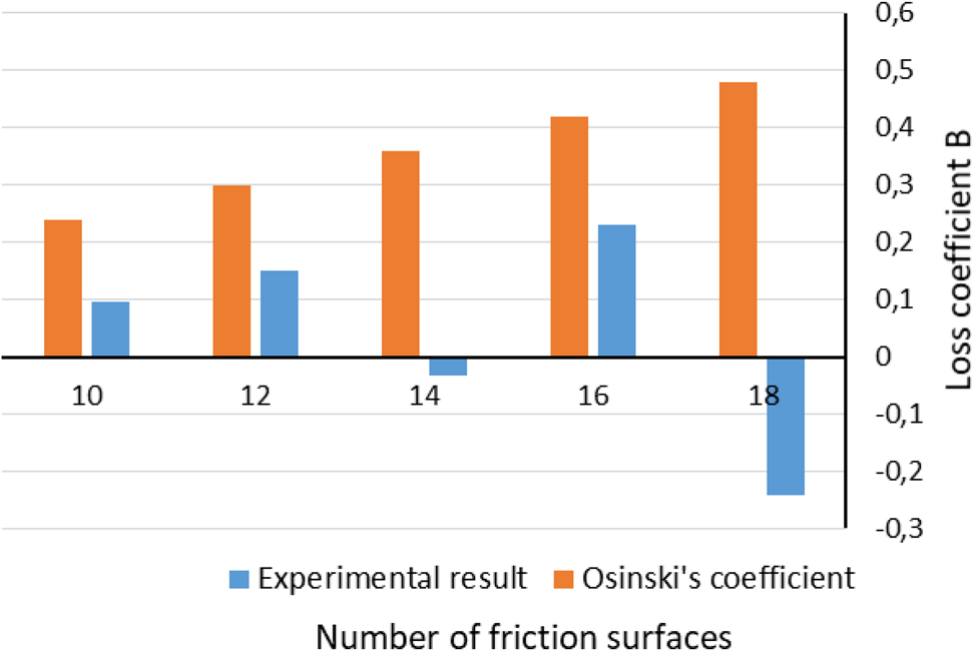
Comparison of experimentally obtained values of loss coefficient B with Osinski’s coefficients.

Loss coefficient B was calculated by comparison the formula (14) with trend line equations from Fig. 12. Estimated values of B are presented in Fig. 13.

**Figure 14:**
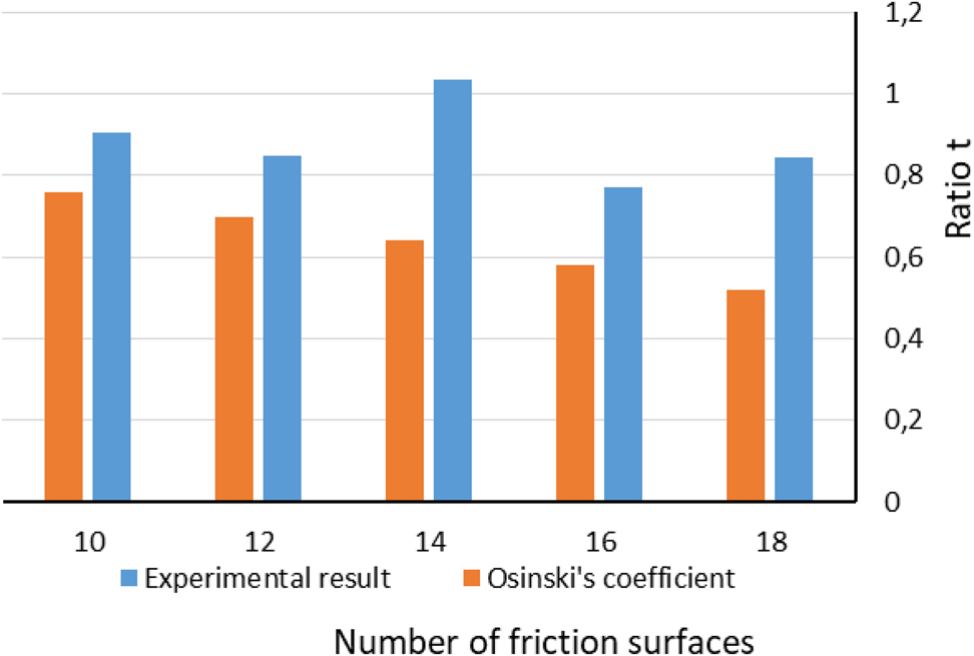
Comparison of experimentally obtained values of coefficient t with Osinski’s coefficients.

In Fig. 14 ratio t obtained according to formula (17) in reference to the number of friction surface is shown. The results are compared to values obtained with application of known Osinski’s coefficients.

### Additional effects appearing during slippage of a clutch

**Figure 15:**
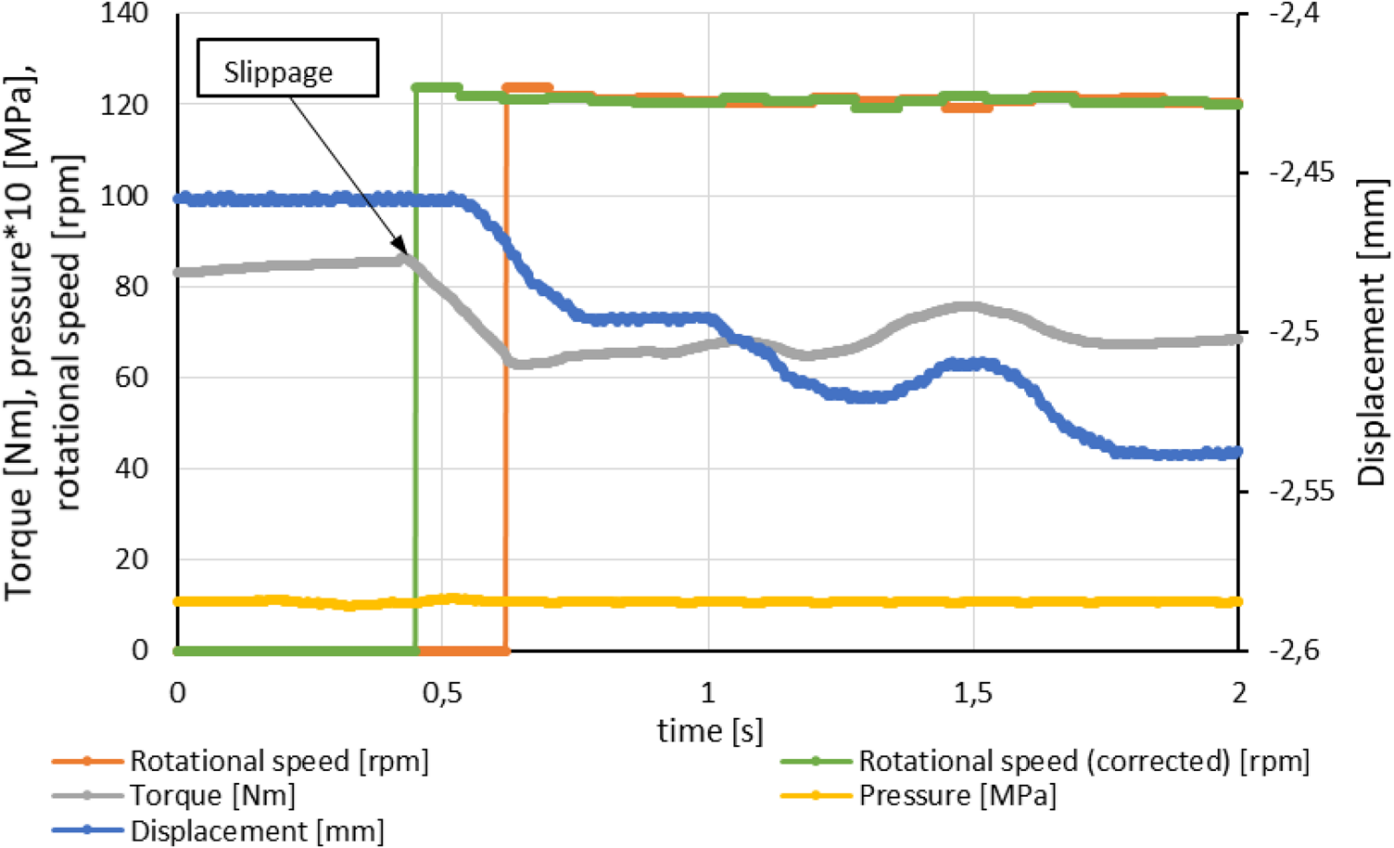
Recorded data of rotational speed, torque, pressure in actuator chamber and displacement presenting increased displacement of discs package after clutch slippage.

**Figure 16:**
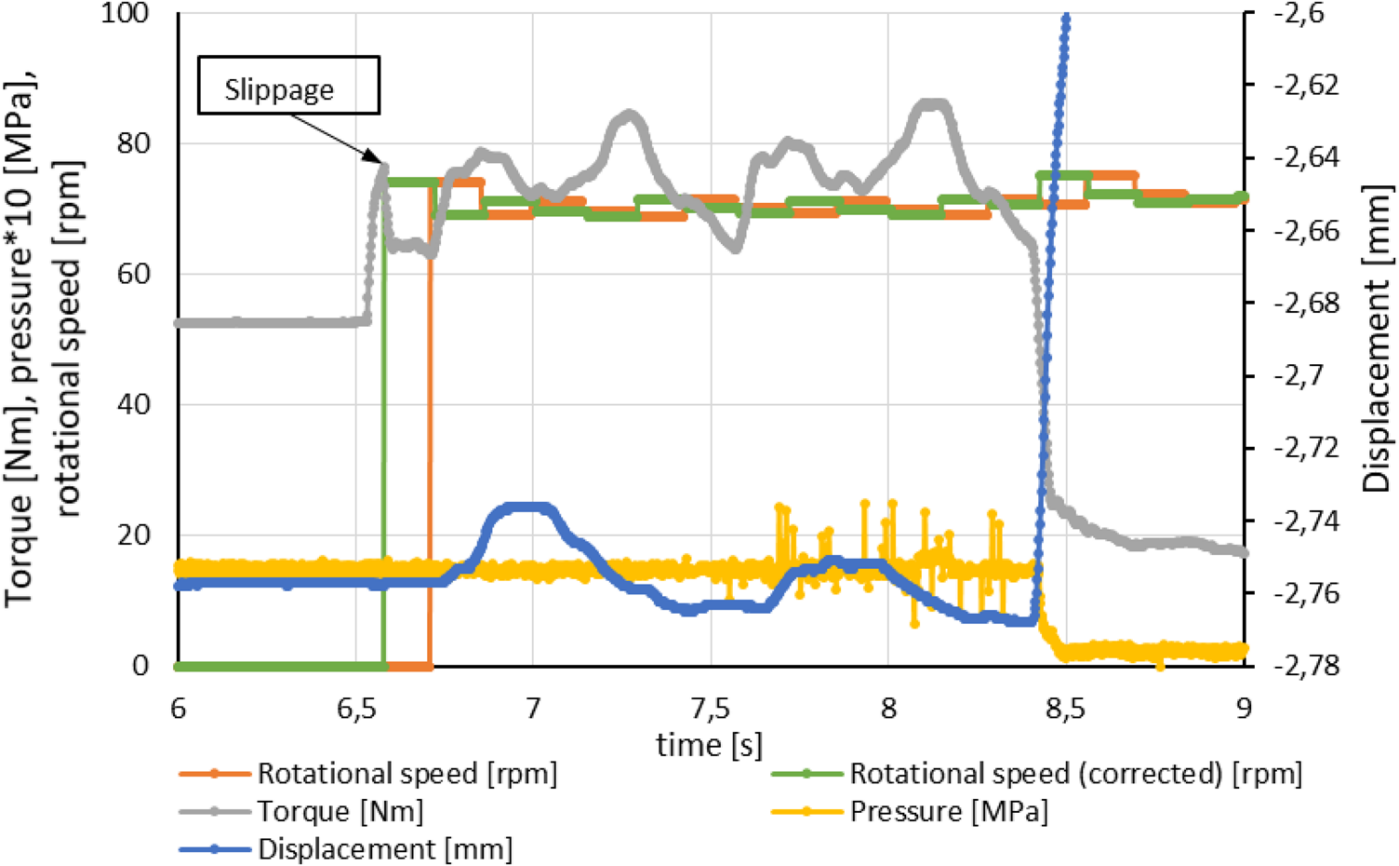
Recorded data of rotational speed, torque, pressure in actuator chamber and displacement presenting decreased displacement of discs package after clutch slippage.

Displacement sensor recorded data indicated that almost every approach of determination of torque capacity has its aftermath. The data shows that shortly after the slippage appeared, the pressure disc moved in the direction of the exerted force or in the opposite direction. Examples of this phenomenon are illustrated in Figs. 15, 16. The graphs show comparison of two different situations. One situation corresponds to increased displacement of discs (Fig. 15) and the other one (Fig. 16) refers to reduced movement. Decreasing values of displacement means that greater displacement of the discs and pressure plate have occurred. While data presented in Fig. 15 were recorded during experiments carried out for 18 friction surfaces, in Fig. 16 are shown results of experiment conducted for 14 friction surfaces. Delay between growth of rotational speed and torque fall is caused by applied speed sensor and objects number that can be detected. To correct the delay additional characteristics named rotational speed (corrected) are shown in Figs. 15 and 16.

### Presentation of discs wear

**Figure 17:**
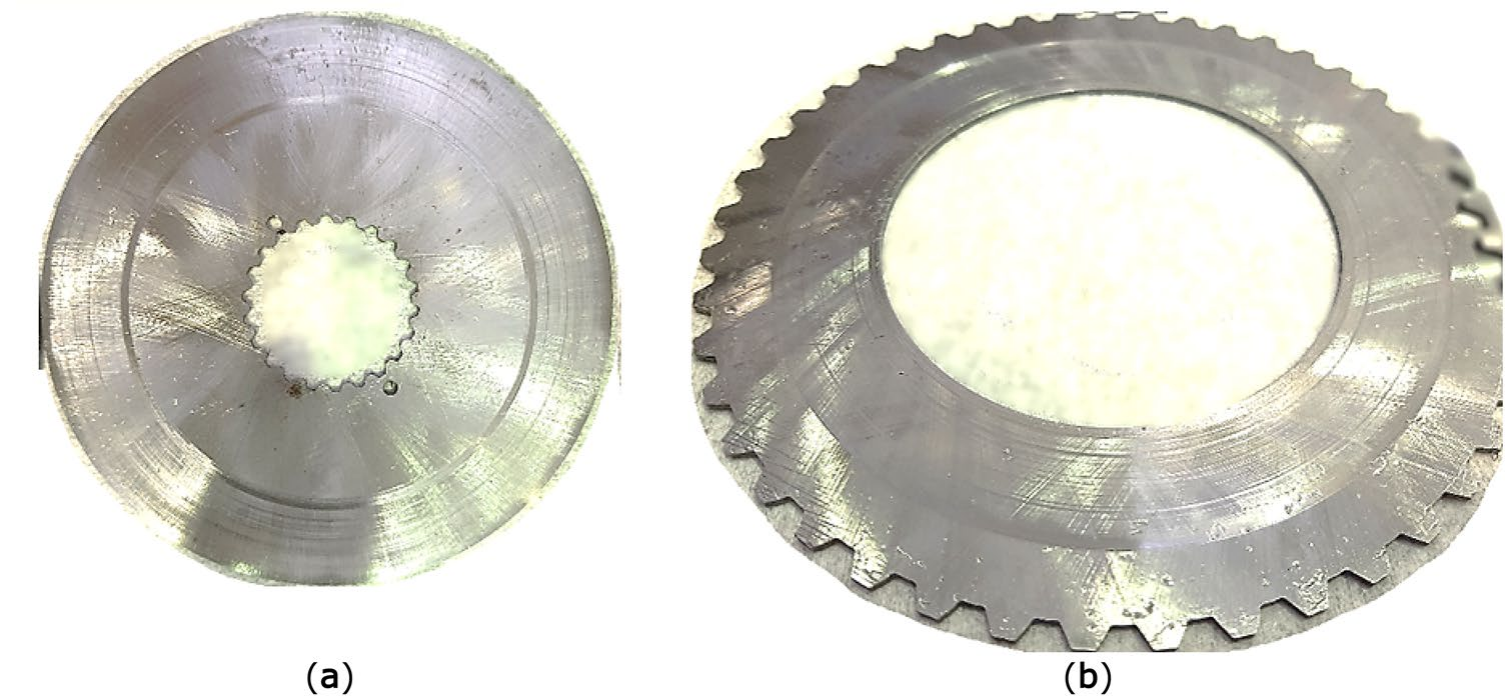
Presentation of wear: (**a**) on friction disc; (**b**) on separator disc.

During experimental tests over two hundred of engagement processes were carried out. As a result of the experiments, friction and separator discs that formed clutch assembly sustained noticeable wear. Pictures illustrated in Fig. 17a,b demonstrate described effect. The friction discs have worn out mostly at annular areas close to internal radius R_i_ and outer radius R_o_ (Fig. 2). These results reaffirm known effect of contact pressure peaks occurring on outer and internal annular areas of friction surfaces^9,13,15^.

## Discussion

According to graphs shown in Fig. 12, it is noticeable that Pearson’s correlation coefficient R obtain values close to one, except for a graph that indicates to 18 contact faces. The lowest value R relates to first serie of tests^26^. Therefore, it is reasonable to claim that methodology is appropriate. For this graph (Fig. 12a) the best-fit line has the biggest differences in reference to collected data, especially within a range of pressure between ca. 0.2 and 0.3 MPa. For approximately the same mean pressure rapidly different torque capacity was reached. It is presumably, because of running-in of adjacent elements on contact faces^26^.

Graphs presented in the chapter 6 show that some variations of approximated lines intersect y axis at graph origin, while other have positive value of vertical intercept (Fig. 12). Furthermore, even for nearly the same clamping forces the torque could greatly vary. Reasons for such significant differences are presumably connected to different contact area, roughness of adjacent surfaces and the contact between discs asperities and grooves. If the normal force is transmitted by small number of asperities and the rest of disc surface is separated from surface of the adjacent disc, then transferred torque might be relatively low. This effect might be additionally intensified when the mechanical contact occurs on small radius in reference to axis of the clutch. Many authors have already proved that pressure peaks appears near to internal diameter of friction surface^13,27,28^. This conclusion is also reaffirmed by experiments described in this article. On the contrary, more uniform pressure distribution or local pressure peaks, which occur further from axis have considerable impact on clutch capacity.

**Figure 18:**
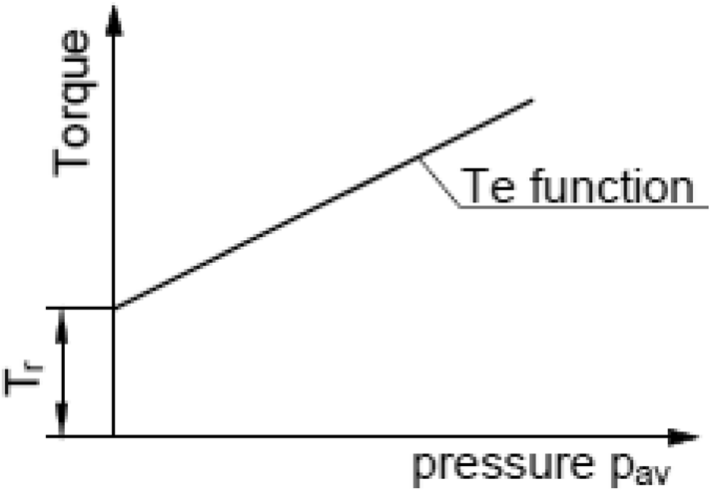
Explanation of minimum (residual) torque transmitted by a clutch.

According to gained data, generated best-fit lines for some variations of clutch package cross y-axis above the origin of coordinate system, as shown schematically in Fig. 18. These values of torque refer to minimum magnitudes of transmitted load that would be called as residual torque. Occurrence of minimum torque T_r_ higher than zero have at least two known causes. First, it may be the result of adhesive and abrasive wear between adjacent discs appearing on contact surfaces^24^. Secondly, as mentioned above, it is also connected to relative angular position between subsequent discs. If asperities of one disc are located inside of grooves of the other disc, this lead to significant growth of torque, even when p_av_ is equal to zero. This effect is related to machining of discs asperities, smoothing of them^23^.

However, according to studies (Fig. 12a,b,d,e), residual torque T_r_ could reach a substantial magnitude of torque estimated to tens of Nm. Especially taking into consideration other values of recorded, transmitted torque which might be smaller even for significant clamping force. Hence, it must be generated by large axial force or be a consequence of another effect, for example at least one of those mentioned previously.

The experimental results and their approximations reach significantly lower magnitudes of losses in relation to literature data (Fig. 14). Values of coefficient B (Fig. 13) below 0 do not have physical basis, at least according to proposed mathematical model. It refers to an effects mentioned previously, concerning running-in contact surfaces, as well as randomly contacted asperities and grooves of adjacent elements. Their influence and prevalence of appearance would be minimalized, if friction and separator discs were made of for example hardened steel^24–26^. During full engagement of the clutch effects relating to thermal differences did not occur, due to static type of experiments^2,3,8^. Nevertheless, assumptions made in mathematical model are not fully fulfilled in any real assembly. Determined values of loss coefficient B involve losses caused by friction on spline connections. The friction depends on torque transmitted by the clutch, as well as distribution of pressure on friction surfaces. Inequality of pressure distribution result in discs tilting, hence, increased friction.

According to data showed in Fig. 16 pressure plate sustain additional movement ca. 0.08 mm in direction of axial force. While for situation in Fig. 17 pressure plate moved in opposite direction about 0.02 mm.

Results of experimental data (Figs. 15, 16) shows abrupt change of torque before the slippage occurrence. This effect happened multiple times during experiments. Torque peak appeared even when pressure relief adjustment was gradually changed. The reason for such effect relates to characteristic and operating performance of gerotor hydraulic motor that drives the test bench. It is caused by mechanical losses in the motor (friction in bearings, friction between cardan shaft with crowned profile and pinion etc.)^29–31^.

Based on analysis of pictures showed in Fig. 17 it is noticeable that higher pressure acted on area closely located to internal radius R_i_. It is proved by significantly decreased wear that occurred on the rest of friction surface^13,27,28^. Some exceptions in form of small dispersed areas appeared near to outer radius R_0_, which contact to sharp edges of friction disc. These edges result in locally appearing contact pressure peaks.

## Conclusions

The paper involves new mathematical model of torque capacity of a multidisc wet clutch and the methodology of performed tests are also described at length. Results of innovative experiments showing influence of number of friction surfaces on torque capacity are presented in the paper. The results show that friction on spline connections in multidisc clutches should not be omitted.

Results of experiments show significantly higher values of torque capacity than values that could be obtained with well-known mathematical model and coefficients proposed by Osinski^10^. Presented information give better insight into relation between clutch capacity and number of discs. As a result, engineers have better opportunity to design such assemblies more accurately. It might allow them to chose better solutions for their systems. Their calculations might result in less overdimensioned assemblies.

Experimental results reveal that approximation of torque transmitted by all tested variations exceeds values estimated with assumption associated with static friction coefficient at its lowest value. Friction on splines was also omitted in the assumption.

For reasons aforementioned, estimation of correction coefficient B was based on maximum value of static friction coefficient^1,10^. Also, if minimum value of friction coefficient would be applied to these analytical calculations, then obtained values of the coefficient B could be negative. Having in mind role of the coefficient and its possible negative value, it could be clearly confusing in reference to information described in chapter 2. Nevertheless, one specified trend line gave such outcome. Presumed explanation refers to running-in and wear of adjacent surfaces. It matches results obtained by Schneider et al.^26^. Although it might be impossible to precisely calculate and measure influence of all components of such complex assembly due to influence of simultaneously occurring effects, such as unevenness of pressure distribution, friction appearing on splines etc.

Further work will be intended to determine influence of friction discs thickness on forces appearing on involute spline connections. Additionally, establishment of more complex and versatile mathematical expression describing this phenomenon should be demanded. Also wider range of friction surfaces and their ratio should be studied. With this purpose in mind, it would be valuable to inspect pressure distribution and thermal deformations of discs on tightness of fits on spline connections. Moreover, future works aimed to study multiple wet clutches and brakes ought to feature friction forces appearing acting on splines.

Following studies will be aimed to extend already acquired data by wider range of friction surfaces that transmit torque.

It would be reasonable to use for further experiments different hydraulic motor with regard to presented issues referring to hydraulic motor,. Such motor should have enhanced performance at rotational speed equal to zero than used GMT-250.

## Data Availability

Three series of measurement of spring force in relation to deflection of disc springs assembled in X configuration. [Data set]. Gdańsk University of Technology. https://doi.org/10.34808/22xd-hs58.
